# Dietary inflammatory and insulinemic potential, risk of hepatocellular carcinoma, and chronic liver disease mortality

**DOI:** 10.1093/jncics/pkad023

**Published:** 2023-03-21

**Authors:** Lu Long, Xing Liu, Jessica Petrick, Wanqing Liu, Jeffrey K Lee, Linda Liao, Michelle J Lai, Wanshui Yang, Towia A Libermann, Lewis R Roberts, Katherine A McGlynn, Fred K Tabung, Xuehong Zhang

**Affiliations:** Channing Division of Network Medicine, Department of Medicine, Brigham and Women’s Hospital, and Harvard Medical School, Boston, MA, USA; Department of Epidemiology and Biostatistics, West China School of Public Health and West China Fourth Hospital, Sichuan University, Chengdu, P.R. China; Department of Nutrition, Harvard T.H. Chan School of Public Health, Boston, MA, USA; Department of Epidemiology, School of Public Health, Fudan University, Shanghai, P.R. China; Slone Epidemiology Center, Boston University, Boston, MA, USA; Department of Pharmaceutical Sciences, Eugene Applebaum College of Pharmacy and Health Sciences, Wayne State University, Detroit, MI, USA; Division of Research, Kaiser Permanente Northern California, Oakland, CA, USA; Department of Gastroenterology, Kaiser Permanente Northern California, San Francisco, CA, USA; Division of Cancer Epidemiology and Genetics, National Cancer Institute, Bethesda, MD, USA; Beth Israel Deaconess Medical Center, Harvard Medical School, Boston, MA, USA; Channing Division of Network Medicine, Department of Medicine, Brigham and Women’s Hospital, and Harvard Medical School, Boston, MA, USA; Department of Nutrition, School of Public Health, Anhui Medical University, Hefei, Anhui, P.R. China; Department of Medicine, Beth Israel Deaconess Medical Center, and Harvard Medical School, Boston, MA, USA; Division of Gastroenterology and Hepatology, Mayo Clinic College of Medicine and Science, Rochester, MN, USA; Division of Cancer Epidemiology and Genetics, National Cancer Institute, Bethesda, MD, USA; Department of Nutrition, Harvard T.H. Chan School of Public Health, Boston, MA, USA; The Ohio State University College of Medicine and Comprehensive Cancer Center, Columbus, OH, USA; Channing Division of Network Medicine, Department of Medicine, Brigham and Women’s Hospital, and Harvard Medical School, Boston, MA, USA; Department of Nutrition, Harvard T.H. Chan School of Public Health, Boston, MA, USA

## Abstract

**Background:**

Diet modulates inflammation and insulin response and may be an important modifiable factor in the primary prevention of hepatocellular carcinoma (HCC) and chronic liver disease (CLD). We developed the empirical dietary inflammatory pattern (EDIP) and empirical dietary index for hyperinsulinemia (EDIH) scores to assess the inflammatory and insulinemic potentials of diet. We prospectively examined the associations of EDIP and EDIH at baseline with the following HCC risk and CLD mortality.

**Design:**

We followed 485 931 individuals in the National Institutes of Health–American Association of Retired Persons Diet and Health Study since 1995. Cox proportional hazards regression was used to calculate multivariable hazard ratios (HRs) and 95% confidence intervals (CIs).

**Results:**

We confirmed 635 incident HCC cases and 993 CLD deaths. Participants in the highest compared with those in the lowest EDIP quartile had a 1.35 times higher risk of developing HCC (95% CI = 1.08 to 1.70, *P*_trend_ = .0005) and a 1.70 times higher CLD mortality (95% CI = 1.41 to 2.04, *P*_trend_ < .0001). For the same comparison, participants with the highest EDIH were at increased risk of HCC (HR = 1.53, 95% CI = 1.20 to 1.95, *P*_trend_ = .0004) and CLD mortality (HR = 1.72, 95% CI = 1.42 to 2.01, *P*_trend_ < .0001). Similar positive associations of scores with HCC risk and CLD mortality were observed for both women and men. Moreover, individuals in both the highest EDIP and EDIH tertiles had a 92% increased HCC risk (95% CI = 1.43 to 2.58) and 98% increased CLD mortality (95% CI = 1.27 to 3.08) compared with those in both lowest tertiles.

**Conclusions:**

Our findings suggest that inflammation and hyperinsulinemia are potential mechanisms linking diet to HCC development and CLD mortality.

Liver cancer is the third-leading cause of cancer-related death worldwide (1). Hepatocellular carcinoma (HCC) accounts for more than 85% of primary liver cancer (2). In the United States, the incidence of liver cancer has tripled since the early 1980s, and it is estimated that 41 260 individuals will be diagnosed in 2022 (3). Approximately 70%-90% of liver cancer arises among persons with chronic liver disease (CLD) (4). Well-established risk factors for HCC and CLD include heavy alcohol consumption, chronic infections with hepatitis B virus (HBV) and hepatitis C virus (HCV), rare monogenic syndromes, aflatoxin, obesity, and type 2 diabetes mellitus (T2D) (5,6). Approximately 35% of HCC cannot be explained by these established risk factors (7).

Mounting evidence suggests an important role of inflammation, insulin resistance, and hyperinsulinemia in HCC development. For example, obesity, a state of low-grade chronic inflammation (8), and T2D, characterized by insulin resistance, impaired insulin secretion, and hyperglycemia (9), have been associated with HCC risk (10,11). In addition, persons with CLD in the setting of metabolic syndrome, such as T2D and obesity, are at higher risk for disease progression (11,12). Epidemiological studies have reported statistically significant associations between prediagnostic levels of widely used inflammation or insulin response biomarkers, such as interleukin-6 (IL-6), C-reactive protein (CRP), tumor necrosis factor (TNF)-α, and HCC risk (13,14) and CLD mortality (15-17). Higher adherence to the T2D prevention diet, Alternative Healthy Eating Index-2010 (AHEI-2010), or Mediterranean diet and lower adherence to the Western diet have been reported to be associated with reduced risk of HCC (18-20) as well as with progression of CLD (20-23). Although these dietary patterns are somewhat associated with inflammation and insulin response, they may not fully capture an individual’s dietary inflammatory and insulinemic potential.

Recently, a food-based empirical dietary inflammatory pattern (EDIP) score (24) and an empirical dietary index for hyperinsulinemia (EDIH) score (25) were developed and validated to assess the ability of long-term dietary patterns to contribute to chronic systemic inflammation and sustained insulin hypersecretion. The EDIP is the weighted sum of 9 antiinflammatory and 9 proinflammatory food groups. The EDIH comprises 18 food groups; 13 were positively associated with C-peptide, and 5 were inversely associated. We hypothesize that reducing a diet’s high inflammatory and hyperinsulinemic potential may influence liver cancer development and CLD progression. To date, the only study on this topic showed a positive association between EDIP or EDIH and risk of HCC (26). This study was based on 142 HCC cases. Because inflammation, insulin resistance, and hyperinsulinemia directly contribute to CLD development and prognosis (27,28), EDIP or EDIH has also been hypothesized to play a role in CLD. However, no study to our knowledge has yet examined the association between EDIP or EDIH and CLD mortality. Therefore, we investigated the association of the EDIP and EDIH as well as their joint associations with risk of HCC based on more than 600 HCC cases and conducted this first study, to our knowledge, on CLD mortality using data from the National Institutes of Health (NIH)-American Association of Retired Persons (AARP) Diet and Health Study.

## Methods

### Study population

The NIH-AARP Diet and Health Study, previously described in detail (29), was begun in 1995-1996. A baseline questionnaire was mailed to AARP members who were 50 to 71 years old and resided in 6 US states (California, Florida, Louisiana, New Jersey, North Carolina, and Pennsylvania) and 2 metropolitan areas (Atlanta, Georgia, and Detroit, Michigan). A total of 566 398 individuals (339 666 men and 226 732 women) satisfactorily completed the baseline questionnaire. In this study, we excluded participants who had 1) cancer at baseline (n = 50 118), 2) extreme caloric intakes (<800 or >4000 for men; <500 or >3500 for women; n = 29 769), and 3) no follow-up time (n = 580). Thus, the final study included 485 931 participants (290 621 men and 195 310 women). The study was approved by the National Cancer Institute Special Studies Institutional Review Board. Informed consent was obtained from all participants.

### Assessment of diet and derivation of EDIP and EDIH scores

Information on diet was collected via a 124-item food-frequency questionnaire (FFQ). The FFQ was validated among 2053 NIH-AARP participants within a measurement error model using 2 nonconsecutive 24-hour dietary recalls as the reference (30). Energy-adjusted correlation coefficients ranged from 0.36 to 0.76 for the 26 nutrient constituents examined (30). EDIP and EDIH scores were developed and validated to reflect the effects of long-term (habitual) diet on inflammation and insulin response (insulin resistance or hyperinsulinemia), respectively (24,31). Briefly, EDIP was derived based on 39 predefined food groups from FFQs using reduced-rank regression followed by stepwise linear regression models to identify a dietary pattern most predictive of 3 inflammatory biomarkers (ie, IL-6, CRP, and TNF-α receptor-2). Likewise, EDIH was derived based on 39 predefined food groups in a separate study to identify a dietary pattern most predictive of C-peptide (an indicator of insulin secretion). A total of 18 foods and food groups were included in each dietary pattern (Supplementary Table 1, available online), which were weighted by the regression coefficients derived from the Nurses’ Health Study cohort and summed across the 18 foods to derive an overall score for the inflammatory or insulinemic potential of the diet of each study participant (24,31). Higher scores reflect more proinflammatory or more hyperinsulinemic dietary patterns.

### Identification of incident HCC cases and liver disease mortality

#### Incident HCC cases

Incident cancer cases were identified through linkage to 11 state cancer registries through December 31, 2011, the most recent follow-up in this AARP study. This approach has been estimated to have a sensitivity of 90% and a specificity of nearly 100% (29). HCC cases were identified using the International Classification of Diseases for Oncology, 3rd edition (ICD-O-3) topography (C22) and morphology codes (8170-8175 for HCC).

#### Deaths from CLDs

The National Death Index Plus was used to ascertain causes of death as of December 31, 2011. Consistent with previous studies (20), deaths from CLDs included deaths as a result of liver fibrosis, alcoholic liver diseases, chronic hepatitis, and cirrhosis (ICD-9: 571.0, 571.2–571.6, 571.8, 571.9; ICD-10: K70, K73, and K74). Liver cancer deaths were not counted in CLD deaths. The National Death Index Plus classification of CLD was validated using the electronic medical records of Kaiser Permanente Medical Care Program members from Northern California and was found to have a specificity of 89% (32).

### Assessment of other covariates

The baseline questionnaire contained information on a broad range of covariates, including demographic characteristics, such as age, race, and ethnicity; level of education; lifestyle factors, such as smoking history, physical activity, and alcohol intake; body weight and height; and medical history, such as diabetes. Body mass index (BMI) was calculated as weight in kilograms divided by the square of height in meters (kg/m^2^). Highest education level was divided into the following groups: less than 8 years, 8-11 years, 12 years or completed high school, post high school training other than college, some college, college graduate, post graduate. The frequency of physical activity was calculated as physical activity at work or home in the past 12 months of at least 20 minutes that resulted in increased breathing or heart rate or sufficient to produce sweating. Reported frequency and quantity of alcohol intake was converted to grams per day. Individuals reported whether they had ever smoked 100 cigarettes or more in their lifetime and whether they currently smoked or had stopped smoking. Dietary information including total calories was obtained using a Quantitative Food Frequency Questionnaire designed for use in this multiethnic population.

#### HBV and HCV infection

Data on chronic HBV and HCV infection status were not available for the participants in this NIH-AARP Diet and Health Study. Based on data from the National Health and Nutrition Examination Survey (NHANES), we investigated the associations of EDIP or EDIH with HBV or HCV status. NHANES is a cross-sectional survey designed to assess the health and nutritional status of the entire US population by enrolling a nationally representative sample of approximately 5000 participants per year. The EDIP and EDIH index have already been developed and validated in the NHANES (33,34).

Because CRP and leucocyte count were simultaneously measured in NHANES in cycles 2001-2002, 2003-2004, 2005-2006, 2007-2008, 2009-2010, 2015-2016, and 2017-2018, we used participants enrolled from these 7 cycles to examine the association of EDIP with HBV and HCV status. Fasting insulin and C-peptide were simultaneously measured in cycle 2001-2002 and 2003-2004, and we used participants enrolled from these 2 cycles to examine the association of EDIH with HBV and HCV status.

### Statistical analyses

HCC person-years were calculated from baseline to the date of primary HCC diagnosis, date of death, or end of follow-up, whichever came first. CLD person-years were calculated from baseline to the date of CLD death, date of death outside CLD, or end of follow-up, whichever came first. We used Cox proportional hazards regression models to estimate hazard ratios (HRs) and 95% confidence intervals (CIs) for different categories of dietary pattern scores. Tests of linear trend across dietary score categories were conducted using the median of each category of dietary pattern scores as a continuous variable. We tested the proportional hazards assumption by using the log (time) cross-product term and the categorical dietary pattern scores as a continuous variable; no violations were observed (*P* > .05).

Based on our a priori assumptions on the relationships between confounders, intermediate variables, exposure, and outcome variables (24-26), we included age at baseline, level of education, race, alcohol use, tobacco smoking, physical activity, aspirin use, self-reported history of diabetes, and total energy intake as a priori covariates in multivariable models. We did not control for BMI in the primary analyses because BMI is a potential intermediate in the association of inflammatory and insulinemic potential of diet with HCC risk and CLD mortality. We adjusted for BMI in sensitivity analyses. Less than 4% of the cohort participants had missing data for a particular covariate. We created an indicator variable for missing and adjusted for it in the multivariable models. The use of the “indicator method” is known to be biased, even in the case of completely random missingness. However, this is most likely not a problem because only 4% had missing data in our study. Subgroup analyses were performed for the categories of age, BMI, alcohol drinking, tobacco smoking, physical activity, and self-reported history of diabetes. The Wald test was used to test for interaction between the empirical dietary indices (continuous) and stratification variables. Lastly, we examined joint associations of EDIP (tertiles) and EDIH (tertiles) with HCC risk and CLD mortality. In sensitivity analyses, cases diagnosed within the first 2 or 5 years of follow-up were separately excluded to address the possibility of reverse causation. We also restricted the study population to self-evaluated excellent health status and included both EDIP and EDIH in the same model in sensitivity analyses. The association between EDIP or EDIH (continuous scale) with HBV and HCV status was assessed using logistic regression models adjusted for age (21 to <40 years, 40 to <60 years, 60 to <80 years, ≥80 years), sex, education (less than high school education, completion of high school, some post high school training, and completion of college), and income (<25 000, 25 000 to <45 000, 45 000 to <75 000, and ≥75 000 dollars per year); we report the Wald *P* value from these analyses. All analyses were performed using SAS 9.4 software (SAS Institute Inc., Cary, NC, USA).

## Results

Participants with higher EDIP or EDIH scores reported lower physical activity and higher BMI, were less educated, and were more likely to have T2D (Table 1). Similar patterns were observed for women (Supplementary Table 2, available online) and men (Supplementary Table 3, available online).

**Table 1.** Age-adjusted characteristics of participants according to quartile categories of the empirical dietary inflammatory pattern (EDIP) and empirical dietary index for hyperinsulinemia (EDIH) at baseline in the National Institutes of Health-American Association of Retired Persons Diet Health Study

	EDIP					EDIH			
	Quartile 1	Quartile 2	Quartile 3	Quartile 4		Quartile 1	Quartile 2	Quartile 3	Quartile 4
	(n = 121 458)	(n = 123 679)	(n = 122 080)	(n = 118 714)		(n = 120 657)	(n = 124 462)	(n = 121 122)	(n = 119 690)
Mean age at baseline (SD), y^a^	61.4 (5.3)	61.8 (5.3)	61.9 (5.3)	61.1 (5.5)		61.8 (5.3)	61.9 (5.3)	61.7 (5.4)	60.7 (5.5)
Sex, %									
Female	44.9	43.1	39.9	32.6		44.0	45.6	41.1	30.0
Male	55.1	56.9	60.1	67.4		56.0	54.4	58.9	70.0
Race, %									
Non-White	6.0	6.0	7.8	9.3		7.5	7.6	7.5	6.4
White	94.0	94.0	92.2	90.7		92.5	92.4	92.5	93.6
College education, %	44.8	41.0	38.5	34.7		47.1	41.2	37.7	33.2
Mean BMI (SD), kg/m^2^	26.4 (4.8)	26.8 (4.9)	27.1 (5)	28 (5.4)		26 (4.5)	26.6 (4.8)	27.3 (5)	28.4 (5.4)
Physical activity at least 20 min ≥5 times per wk, %	23.2	19.2	17.8	17.3		25.9	19.6	16.4	15.5
Alcohol, mean drinks per wk (SD)	8.4 (15.5)	5.4 (10.5)	4.4 (10.1)	3.8 (11.2)		9 (16.8)	5 (10.8)	4.1 (9.3)	4 (9.4)
Current smoking, %	14.3	12.6	10.9	11.1		11.4	11.7	11.9	13.9
History of diabetes, %	6.5	7.1	8.8	13.7		5.0	6.4	9.0	15.9
Aspirin use, %	74.0	74.2	73.0	71.2		73.7	73.3	72.7	72.7
Total energy intake, mean (SD) kcal/d	1869 (672)	1716 (633)	1698 (637)	1902 (714)		1969 (667)	1683 (612)	1632 (632)	1900 (707)

a Values are not age adjusted. Values are standardized to the age distribution of the study population. BMI = body mass index.

After a median follow-up of 15.5 years, a total of 635 incident HCCs were identified. In the multivariable analyses, compared with participants in the lowest EDIP quartile, those in the highest EDIP quartile had a 1.35 times higher risk of developing HCC (95% CI = 1.08-1.70, *P*_trend_ = .0005) (Table 2). Those in the highest EDIH had a 1.53 times higher risk of HCC compared with those in the lowest EDIH quartile (95% CI = 1.20-1.95, *P*_trend_ = .0004) (Table 2). The positive associations of each dietary pattern score with HCC risk were observed for both women (Supplementary Table 4, available online) and men (Supplementary Table 5, available online).

**Table 2.** Hazard ratios (HRs) and 95% confidence intervals (CIs) for hepatocellular carcinoma (HCC) and chronic liver disease (CLD) mortality by quartiles of empirical dietary inflammatory pattern (EDIP) and empirical dietary index for hyperinsulinemia (EDIH) at baseline in the National Institutes of Health-American Association of Retired Persons Diet Health Study

	Quartile 1 HR (95% CI)	Quartile 2 HR (95% CI)	Quartile 3 HR (95% CI)	Quartile 4 HR (95% CI)	*P*_trend_ ^d^
EDIP					
HCC					
No. of cases	134	116	175	210	
Age-adjusted model^a^	1 (Referent)	0.83 (0.64 to 1.06)	1.23 (0.98 to 1.54)	1.52 (1.22 to 1.89)	<.0001
Model 1^b^	1 (Referent)	0.87 (0.67 to 1.12)	1.25 (0.99 to 1.58)	1.48 (1.18 to 1.87)	<.0001
Model 2^c^	1 (Referent)	0.87 (0.67 to 1.12)	1.23 (0.97 to 1.55)	1.35 (1.08 to 1.70)	.0005
CLD mortality					
No. of cases	201	216	250	326	
Age-adjusted model^a^	1 (Referent)	1.04 (0.86 to 1.26)	1.21 (1.01 to 1.46)	1.67 (1.40 to 2.00)	<.0001
Model 1^b^	1 (Referent)	1.21 (1.00 to 1.48)	1.39 (1.14 to 1.68)	1.84 (1.53 to 2.21)	<.0001
Model 2^c^	1 (Referent)	1.20 (0.99 to 1.47)	1.35 (1.11 to 1.63)	1.70 (1.41 to 2.04)	<.0001
EDIH					
HCC					
No. of cases	116	139	169	211	
Age-adjusted model^a^	1 (Referent)	1.19 (0.93 to 1.52)	1.47 (1.16 to 1.86)	1.82 (1.45 to 2.29)	<.0001
Model 1^b^	1 (Referent)	1.24 (0.96 to 1.60)	1.45 (1.13 to 1.86)	1.81 (1.42 to 2.30)	<.0001
Model 2^c^	1 (Referent)	1.22 (0.94 to 1.57)	1.37 (1.07 to 1.76)	1.53 (1.20 to 1.95)	.0004
CLD mortality					
No. of cases	193	233	249	318	
Age-adjusted model^a^	1 (Referent)	1.19 (0.98 to 1.44)	1.32 (1.09 to 1.59)	1.76 (1.47 to 2.10)	<.0001
Model 1^b^	1 (Referent)	1.42 (1.16 to 1.73)	1.58 (1.29 to 1.93)	1.93 (1.59 to 2.35)	<.0001
Model 2^c^	1 (Referent)	1.40 (1.15 to 1.71)	1.52 (1.24 to 1.86)	1.72 (1.42 to 2.10)	<.0001

a Adjusted for age (in years).

b Adjusted for age (in years), sex (women, men), race (White, Non-White), education (≤11 years, high school, vocational technology school, some college, college or postgraduate), physical activity (never, rarely, 1-3 times per month, 1-2 times per week, 3-4 times per week, ≥5 times per week), smoking status (never, past, current), aspirin use (yes, no), alcohol intake (g/d, continuous), and total calorie intake (kcal/d, continuous).

c Adjusted for covariates in Model 1 plus history of diabetes (yes, no).

d The *P*_trend_ was obtained using EDIP or EDIH quartile medians as an ordinal variable adjusted for the covariates listed above.

A total of 993 CLD deaths were identified. Compared with participants in the lowest EDIP quartile, those in the highest EDIP quartile had a 1.70 times higher CLD mortality (95% CI = 1.41 to 2.04, *P*_trend_ < .0001). Those in the highest EDIH quartile had a 1.72 times higher risk of CLD compared with those in the lowest EDIH quartile (95% CI = 1.42 to 2.10, *P*_trend_ < .0001) (Table 2). The positive associations of each dietary pattern score with CLD mortality were also observed for both women (Supplementary Table 4, available online) and men (Supplementary Table 5, available online), although the associations appeared slightly stronger in women.

**Figure 1.**
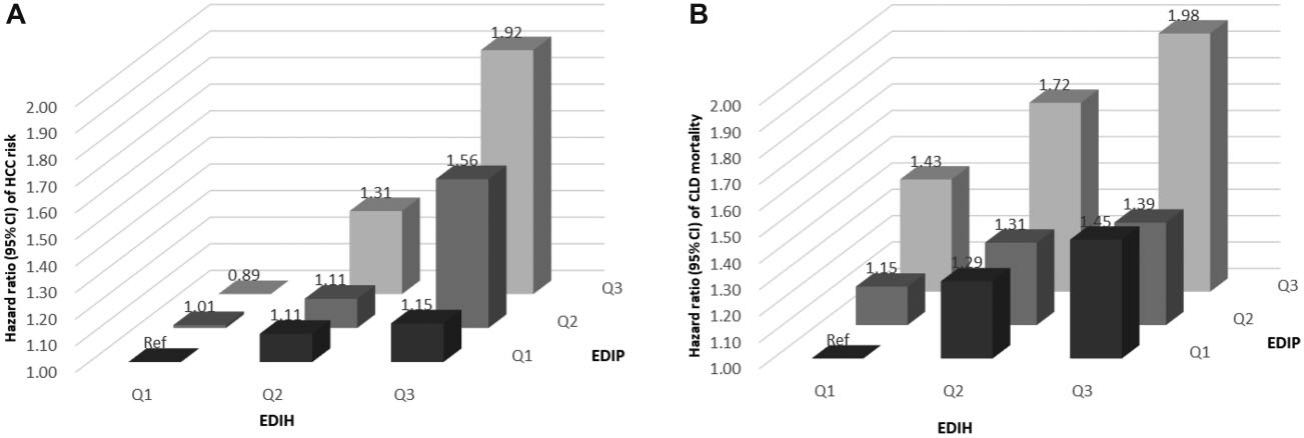
Joint associations of empirical dietary inflammatory pattern (EDIP) and empirical dietary index for hyperinsulinemia (EDIH) at baseline with risk of hepatocellular carcinoma (HCC) (**A**) and chronic liver disease (CLD) mortality (**B**). Hazard ratios were calculated in Cox proportional hazard models after adjustment for age (in years), sex (women, men), race (White, Non-White), education (≤11 years, high school, vocational technology school, some college, college or postgraduate), physical activity (never, rarely, 1-3 times per month, 1-2 times per week, 3-4 times per week, ≥5 times per week), smoking status (never, past, current), aspirin use (yes, no), alcohol intake (g/d, continuous), total calorie intake (kcal/d, continuous), and history of diabetes (yes, no). CI = confidence interval.

In subgroup analysis, we observed similar associations of EDIP and EDIH with risk of HCC and CLD mortality (Tables 3-6). In the joint analysis of EDIP and EDIH, individuals in both the highest EDIP and EDIH tertiles had 92% (95% CI = 1.43% to 2.58%) increased risk of HCC and 98% (95% CI = 1.27% to 3.08%) increased CLD mortality compared with those in both lowest tertiles (Figure 1). In sensitivity analyses, we excluded HCC cases identified within the first 2 or first 5 years of follow-up; only very few cases were excluded, and the results were essentially unchanged (Supplementary Table 6, available online). Additional adjustments for BMI or mutual adjustment for these 2 dietary patterns in the same model did not materially change the associations either (Supplementary Table 7, available online). Results for EDIP and EDIH were similar after we restricted the study population to self-evaluated excellent health status (data not shown).

**Table 3.** Stratified analyses for association between empirical dietary inflammatory pattern at baseline with risk of hepatocellular carcinoma in the National Institutes of Health-American Association of Retired Persons Diet Health Study^a^

	Quartile 1 HR (95% CI)	Quartile 2 HR (95% CI)	Quartile 3 HR (95% CI)	Quartile 4 HR (95% CI)	*P*_interaction_ *^b^*
Age, y					.08
<60 (n = 167)	1 (Referent)	1.49 (0.87 to 2.57)	1.87 (1.11 to 3.14)	2.08 (1.27 to 3.40)	
≥60 (n = 468)	1 (Referent)	0.75 (0.56 to 1.00)	1.10 (0.85 to 1.43)	1.11 (0.85 to 1.44)	
BMI, kg/m^2^					.66
<25 (n = 135)	1 (Referent)	1.06 (0.64 to 1.73)	0.99 (0.60 to 1.66)	1.35 (0.83 to 2.20)	
≥25 (n = 483)	1 (Referent)	0.81 (0.60 to 1.10)	1.34 (1.02 to 1.75)	1.32 (1.01 to 1.72)	
Smoking					.004
Ever (n = 449)	1 (Referent)	0.74 (0.55 to 0.98)	1.06 (0.81 to 1.37)	1.07 (0.82 to 1.38)	
Never (n = 169)	1 (Referent)	1.93 (1.04 to 3.59)	2.50 (1.39 to 4.51)	2.83 (1.60 to 5.01)	
Type 2 diabetes mellitus					.07
Yes (n = 169)	1 (Referent)	0.85 (0.63 to 1.15)	1.35 (1.03 to 1.76)	1.36 (1.04 to 1.79)	
No (n = 466)	1 (Referent)	0.90 (0.55 to 1.46)	0.86 (0.53 to 1.38)	1.05 (0.68 to 1.61)	
Alcohol, drinks per wk					
0 (n = 69)	1 (Referent)	0.64 (0.27 to 1.51)	1.21 (0.60 to 2.47)	1.19 (0.60 to 2.37)	.62
<3 (n = 493)	1 (Referent)	0.81 (0.61 to 1.09)	1.16 (0.88 to 1.51)	1.14 (0.87 to 1.48)	
≥3 (n = 73)	1 (Referent)	1.42 (0.73 to 2.76)	1.20 (0.59 to 2.46)	1.88 (1.00 to 3.56)	
Physical activity, times per wk					.39
<1 (n = 240)	1 (Referent)	0.92 (0.59 to 1.45)	1.32 (0.88 to 1.98)	1.48 (1.01 to 2.18)	
≥1 (n = 395)	1 (Referent)	0.86 (0.63 to 1.17)	1.20 (0.90 to 1.60)	1.20 (0.90 to 1.60)	

a All models were stratified for age (in years), sex (women, men), race (White, Non-White), education (≤11 years, high school, vocational technology school, some college, college or postgraduate), physical activity (never, rarely, 1-3 times per month, 1-2 times per week, 3-4 times per week, ≥5 times per week), smoking status (never, past, current), aspirin use (yes, no), alcohol intake (g/d, continuous), total calorie intake (kcal/d, continuous), and history of diabetes (yes, no). Of note, variables examined in this table were not adjusted. BMI = body mass index; CI = confidence interval; HR = hazard ratio.

b The *P*_interaction_ was calculated using the likelihood ratio test comparing models with and without the interaction term.

**Table 4.** Stratified analyses for association between empirical dietary inflammatory pattern at baseline with chronic liver disease mortality in the National Institutes of Health-American Association of Retired Persons Diet Health Study^a^

	Quartile 1 HR (95% CI)	Quartile 2 HR (95% CI)	Quartile 3 HR (95% CI)	Quartile 4 HR (95% CI)	*P*_interaction_ ^b^
Age, y					.90
<60 (n = 261)	1 (Referent)	1.19 (0.80 to 1.77)	1.62 (1.11 to 2.35)	1.85 (1.30 to 2.64)	
≥60 (n = 732)	1 (Referent)	1.19 (0.95 to 1.50)	1.24 (0.99 to 1.56)	1.58 (1.27 to 1.97)	
BMI, kg/m^2^					.14
<25 (n = 285)	1 (Referent)	1.86 (1.30 to 2.64)	1.49 (1.02 to 2.17)	2.17 (1.51 to 3.12)	
≥25 (n = 679)	1 (Referent)	0.98 (0.77 to 1.25)	1.20 (0.95 to 1.51)	1.49 (1.20 to 1.86)	
Smoking					.05
Ever (n = 709)	1 (Referent)	1.24 (1.00 to 1.55)	1.31 (1.05 to 1.63)	1.57 (1.27 to 1.95)	
Never (n = 233)	1 (Referent)	1.00 (0.64 to 1.57)	1.31 (0.87 to 1.98)	1.75 (1.19 to 2.58)	
Type 2 diabetes mellitus					.06
Yes (n = 202)	1 (Referent)	1.29 (1.03 to 1.60)	1.45 (1.17 to 1.80)	1.79 (1.45 to 2.20)	
No (n = 791)	1 (Referent)	0.81 (0.51 to 1.28)	0.85 (0.55 to 1.32)	1.11 (0.75 to 1.64)	
Alcohol, drinks per wk					.32
0 (n = 89)	1 (Referent)	1.22 (0.60 to 2.50)	1.02 (0.49 to 2.10)	1.35 (0.69 to 2.63)	
<3 (n = 655)	1 (Referent)	1.26 (0.98 to 1.61)	1.46 (1.14 to 1.87)	1.77 (1.39 to 2.25)	
≥3 (n = 249)	1 (Referent)	1.17 (0.81 to 1.71)	1.45 (1.01 to 2.10)	2.19 (1.56 to 3.06)	
Physical activity, times per wk					.65
<1 (n = 466)	1 (Referent)	1.10 (0.81 to 1.50)	1.47 (1.10 to 1.96)	1.73 (1.31 to 2.28)	
≥1 (n = 527)	1 (Referent)	1.26 (0.98 to 1.63)	1.22 (0.94 to 1.59)	1.65 (1.29 to 2.12)	

a All models were stratified for age (in years), sex (women, men), race (White, Non-White), education (≤11 years, high school, vocational technology school, some college, college or postgraduate), physical activity (never, rarely, 1-3 times per month, 1-2 times per week, 3-4 times per week, ≥5 times per week), smoking status (never, past, current), aspirin use (yes, no), alcohol intake (g/d, continuous), total calorie intake (kcal/d, continuous) and history of diabetes (yes, no). Of note, variables examined in this table were not adjusted. BMI = body mass index; CI = confidence interval; HR = hazard ratio.

b The *P*_interaction_ was calculated using the likelihood ratio test comparing models with and without the interaction term.

**Table 5.** Stratified analyses for association between empirical dietary index for hyperinsulinemia at baseline with risk of hepatocellular carcinoma in the National Institutes of Health–American Association of Retired Persons Diet Health Study^a^

	Quartile 1 HR (95% CI)	Quartile 2 HR (95% CI)	Quartile 3 HR (95% CI)	Quartile 4 HR (95% CI)	*P*_interaction_ ^b^
Age, y					.25
<60 (n = 167)	1 (Referent)	1.78 (1.00 to 3.16)	1.87 (1.06 to 3.29)	2.05 (1.20 to 3.50)	
≥ 60 (n = 468)	1 (Referent)	1.12 (0.84 to 1.50)	1.25 (0.94 to 1.66)	1.24 (0.94 to 1.64)	
BMI, kg/m^2^					.19
<25 (n = 135)	1 (Referent)	1.15 (0.69 to 1.90)	1.42 (0.85 to 2.35)	1.59 (0.94 to 2.67)	
≥25 (n = 483)	1 (Referent)	1.25 (0.92 to 1.70)	1.34 (1.00 to 1.81)	1.45 (1.09 to 1.92)	
Smoking					.14
Ever (n = 449)	1 (Referent)	1.15 (0.86 to 1.54)	1.21 (0.91 to 1.62)	1.31 (0.99 to 1.73)	
Never (n = 169)	1 (Referent)	1.65 (0.95 to 2.85)	2.07 (1.22 to 3.52)	2.03 (1.19 to 3.45)	
Type 2 diabetes mellitus					.05
Yes (n = 169)	1 (Referent)	1.31 (0.98 to 1.77)	1.54 (1.15 to 2.05)	1.57 (1.17 to 2.09)	
No (n = 466)	1 (Referent)	0.92 (0.55 to 1.54)	0.82 (0.49 to 1.37)	0.95 (0.60 to 1.51)	
Alcohol, drinks per wk					.85
0 (n = 69)	1 (Referent)	1.01 (0.47 to 2.17)	0.80 (0.36 to 1.80)	1.22 (0.59 to 2.54)	
<3 (n = 493)	1 (Referent)	1.31 (0.97 to 1.77)	1.44 (1.08 to 1.94)	1.40 (1.05 to 1.87)	
≥3 (n = 73)	1 (Referent)	0.85 (0.42 to 1.73)	1.28 (0.66 to 2.50)	1.51 (0.79 to 2.90)	
Physical activity, times per wk					.84
<1 (n = 240)	1 (Referent)	1.42 (0.89 to 2.25)	1.67 (1.07 to 2.61)	1.60 (1.04 to 2.45)	
≥1 (n = 395)	1 (Referent)	1.16 (0.85 to 1.58)	1.25 (0.92 to 1.70)	1.37 (1.01 to 1.85)	

a All models were stratified for age (in years), sex (women, men), race (White, Non-White), education (≤11 years, high school, vocational technology school, some college, college or postgraduate), physical activity (never, rarely, 1-3 times per month, 1-2 times per week, 3-4 times per week, ≥5 times per week), smoking status (never, past, current), aspirin use (yes, no), alcohol intake (g/d, continuous), total calorie intake (kcal/d, continuous) and history of diabetes (yes, no). Of note, variables examined in this table were not adjusted. BMI = body mass index; CI = confidence interval; HR = hazard ratio.

b The *P*_interaction_ was calculated using the likelihood ratio test comparing models with and without the interaction term.

**Table 6.** Stratified analyses for association between empirical dietary index for hyperinsulinemia at baseline with chronic liver disease mortality among National Institutes of Health-American Association of Retired Persons Diet Health Study^a^

	Quartile 1 HR (95% CI)	Quartile 2 HR (95% CI)	Quartile 3 HR (95% CI)	Quartile 4 HR (95% CI)	*P*_interaction_ ^b^
Age, y					.26
<60 (n = 261)	1 (Referent)	1.29 (0.85 to 1.95)	1.67 (1.13 to 2.49)	1.64 (1.12 to 2.41)	
≥60 (n = 732)	1 (Referent)	1.39 (1.10 to 1.75)	1.37 (1.08 to 1.74)	1.60 (1.27 to 2.01)	
BMI, kg/m^2^					.08
<25 (n = 285)	1 (Referent)	1.32 (0.92 to 1.89)	1.52 (1.05 to 2.20)	2.16 (1.51 to 3.09)	
≥25 (n = 679)	1 (Referent)	1.35 (1.05 to 1.74)	1.44 (1.12 to 1.84)	1.55 (1.22 to 1.98)	
Smoking					.50
Ever (n = 709)	1 (Referent)	1.45 (1.15 to 1.82)	1.64 (1.30 to 2.07)	1.71 (1.36 to 2.15)	
Never (n = 233)	1 (Referent)	1.16 (0.77 to 1.74)	1.02 (0.67 to 1.55)	1.47 (0.99 to 2.18)	
Type 2 diabetes mellitus					.06
Yes (n = 202)	1 (Referent)	1.25 (1.01 to 1.55)	1.31 (1.06 to 1.63)	1.36 (1.10 to 1.69)	
No (n = 791)	1 (Referent)	0.89 (0.49 to 1.28)	0.96 (0.57 to 1.51)	1.07 (0.86 to 1.64)	
Alcohol, drinks per wk					.85
No drink (n = 89)	1 (Referent)	1.36 (0.63 to 2.94)	1.42 (0.66 to 3.06)	1.15 (0.53 to 2.48)	
<3 (n = 655)	1 (Referent)	1.52 (1.17 to 1.97)	1.46 (1.12 to 1.91)	1.90 (1.47 to 2.44)	
≥3 (n = 249)	1 (Referent)	1.13 (0.79 to 1.63)	1.75 (1.23 to 2.47)	1.49 (1.03 to 2.16)	
Physical activity, times per wk					.84
<1 (n = 466)	1 (Referent)	1.25 (0.91 to 1.72)	1.48 (1.08 to 2.01)	1.60 (1.19 to 2.16)	
≥1 (n = 527)	1 (Referent)	1.45 (1.11 to 1.88)	1.42 (1.08 to 1.86)	1.69 (1.29 to 2.20)	

a All models were stratified for age (in years), sex (women, men), race (White, Non-White), education (≤11 years, high school, vocational technology school, some college, college or postgraduate), physical activity (never, rarely, 1-3 times per month, 1-2 times per week, 3-4 times per week, ≥5 times per week), smoking status (never, past, current), aspirin use (yes, no), alcohol intake (g/d, continuous), total calorie intake (kcal/d, continuous) and history of diabetes (yes, no). Of note, variables examined in this table were not adjusted. BMI = body mass index; CI = confidence interval; HR = hazard ratio.

b The *P*_interaction_ was calculated using the likelihood ratio test comparing models with and without the interaction term.

Among 30 172 NHANES adults from 7 cycles with available data on diet, CRP, leucocyte, and viral hepatitis, 512 individuals were anti-HCV positive and 126 individuals were HBV surface antigen positive. In NHANES, we found no association between EDIP with HBV or HCV status (OR = 0.23, 95% CI = 0.03 to 1.57 for HBsAg positivity; OR = 0.48, 95% CI = 0.15-1.58 for HCV positivity; and OR = 0.46, 95% CI = 0.15-1.46 for HBV/HCV positivity, respectively). Among 15 337 NHANES adults from 2 cycles with available data on diet, fasting insulin, C-peptide, and viral hepatitis, 199 individuals were anti-HCV positive and 41 individuals were HBV surface antigen positive. We found no association between EDIH with HBV or HCV status (*P*_trend_ = .12, .22, and .18 for HBsAg positivity, HCV positivity, and HBV/HCV positivity, respectively).

## Discussion

In this prospective cohort study, we observed statistically significant positive associations between baseline dietary inflammatory or insulinemic potential with HCC incidence and CLD mortality. These associations persisted even after additional adjustment for BMI and T2D.

Several studies have reported that some dietary patterns, including AHEI-2010, the T2D prevention diet, and the Mediterranean diet, are inversely associated with incidence of HCC and CLD mortality (18-23). All of these dietary patterns are associated with inflammation and insulin response. There is evidence that high concentrations of biomarkers of inflammation and insulin resistance directly correlate with the dietary patterns that increase the risk of diseases, including HCC and CLD, and may play key roles in eliciting the biological effects of diet (35,36). Epidemiological studies have reported positive associations of prediagnostic circulating levels of biomarkers of inflammation or insulin response such as CRP, IL-6, TNF-α receptor-2, and C-peptide HCC risk (13,14) and CLD mortality (15-17). These biomarkers serve as mediators of many downstream cancer-related signaling pathways or activate signaling pathways controlling inflammatory, immune, and stress responses, which may cause liver damage–mediated inflammation and carcinogenesis (16,37). Glycemic index (GI), glycemic load (GL), insulin index, and insulin load have been used to directly quantify the postprandial blood glucose and insulin response to a range of common foods (38,39). Nonetheless, GI and GL have limited capacity to account for noncarbohydrate factors that may affect insulin response. The findings on the association of GI and GL with HCC risk were mixed, with positive (40), null (41), and inverse (42) associations reported. On the contrary, insulin index was either not predictive of fasting C-peptide concentrations (insulin exposure) or showed an unexpected inverse association with fasting C-peptide, because insulin index is based on short-term increases in insulin after a meal (31,43).

We previously developed and validated an empirical hypothesis-driven food-based dietary index for hyperinsulinemia and inflammation that were based on habitual diets and strongly predictive of C-peptide and inflammatory markers CRP, IL-6, and TNF-α receptor-2 concentrations (24,25). These dietary indices (ie, EDIP and EDIH) specifically assesses the possible contribution of food to chronic inflammation and hyperinsulinemia, although the general dietary quality is assessed by dietary indices including AHEI, the Dietary Approaches to Stop Hypertension diet, and the alternate Mediterranean Diet. EDIP or EDIH places more emphasis on unusual foods linked to inflammation or hyperinsulinemia and shares few dietary components with other indices (43). For instance, it recommends consuming more antiinflammatory and antihyperinsulinemia foods such as green leafy vegetables and coffee (43).

Previous studies have reported that EDIP and EDIH were associated with an elevated risk of T2D (44,45) and cardiovascular disease (43), which may have overlapping mechanisms with HCC and CLD (10-12). Furthermore, evidence is accumulating for the associations of EDIP or EDIH with cancer risk (46-48). To date, limited study has evaluated the associations of these dietary indices with CLD death. The only study based on data from the Nurses’ Health Study and Health Professionals Follow-up Study examined the association between EDIP or EDIH and HCC risk (26). In a pooled analysis of these 2 cohorts including 142 HCC cases, the EDIP and EDIH were associated with HCC risk, with hazard ratios of 2.03 (95% CI = 1.31 to 3.16, *P*_trend_ = .001) for EDIP and 1.61 (95% CI = 1.06 to 2.43, *P*_trend_ = .02) for EDIH when comparing participants in the top tertile categories with the bottom tertile categories. The magnitude of the associations was attenuated, and EDIH was no longer associated with HCC risk after adjusting for BMI and T2D. In addition, no statistically significant association was found among men in that study. In this study, we showed a robustly positive association between dietary insulinemic potential and risk of HCC and CLD mortality with 635 HCC cases and 993 CLD cases from the NIH-AARP Diet and Health Study. Also, the associations of each dietary pattern score with HCC risk were observed for both women and men. In addition, individuals consuming diets with both higher inflammatory and insulinemic potential had the highest HCC risk and CLD mortality.

Additionally, we observed similar associations of the empirical hypothesis–driven dietary indices with risk of HCC and CLD mortality across different subgroups, although the associations of EDIP with HCC appeared slightly stronger for individuals who smoked. The insulin or insulin-like growth factor signaling pathway is known to be activated in HCC (49). Tobacco smoking can influence systemic concentrations of insulin-like growth factor (50). Our results may suggest the dynamic interplay of behavioral factors with insulinemic and inflammatory potential of diet in the etiology of HCC and liver disease. It is worth noting that the power is limited for most subgroup analyses; therefore, these results should be interpreted with caution. Further studies are needed to confirm these findings and elucidate more specific underlying mechanisms.

Our study has several strengths. The prospective design and long follow-up reduce the potential for reverse causation. Also, our results were essentially unchanged after we excluded the limited cases that occurred 2 or 5 years from baseline. The large sample size and comprehensive covariate data allowed for adjustment of multiple possible confounding factors. This study also has some limitations. First, data on HBV and HCV status were not available in this study, although the estimated prevalence was 0.1% for HBV and 1.0% for HCV in the general US population (51,52). We evaluated the associations between EDIP or EDIH and HBV or HCV status in the NHANES and found no correlations. Furthermore, previous studies of liver cancer risk with BMI (53), fish intake (54), and coffee (55) reported no major change in results with or without adjustment for HBV or HCV. Therefore, the relationship between diet and HCC risk is unlikely to be statistically significantly confounded by HBV or HCV infection status. Second, diet and other covariates from questionnaires were self-reported and were subject to measurement errors, which may have resulted in misclassification of exposure. However, the FFQ used to measure dietary intake was validated (30). Third, as an observational study, residual or unknown confounding cannot be completely ruled out. In addition, we lacked data on liver diseases. Because the vast majority of liver cancer arises from liver diseases (56), when examining associations with liver cancer risk, it is generally agreed not to adjust for liver diseases (ie, a causal intermediate factor). Thus, our proposed study can validly address important hypotheses even without data on liver disease that are intermediate to exposure–disease. Nonetheless, because individuals with liver disease, such as those with liver cirrhosis, usually do not report their health as excellent, very good, or good, we restricted the analysis to self-evaluated excellent health status; our findings were essentially unchanged. Fourth, the study participants were predominantly White; therefore, generalizability of our findings to other racial or ethnic groups may be limited. Fifth, we had only baseline data and could not evaluate the changes of diet during follow-up. In our study, a 124-item FFQ was used to attain data on the habitual diet. The FFQ was validated among 2053 NIH-AARP participants within a measurement error model using 2 nonconsecutive 24-hour dietary recalls as the reference. The performance of the FFQ in the NIH-AARP Diet and Health Study (30), in conjunction with the study’s large sample size and wide range of dietary intake, is likely to allow detection of moderate relative risks between many energy-adjusted nutrients and common cancers. Finally, the outcomes of interest in this analysis were the incidence of liver cancer and CLD mortality. Although it would be ideal to study the incidence of CLD in addition to morality, there is no CLD registry in the United States and no single medical record system to identify the diagnosis of CLD. Therefore, CLD mortality was analyzed in the current study. Clearly, investigating dietary etiologies of CLD incidence is warranted.

In conclusion, we found that higher dietary inflammatory or insulinemic potential was associated with an increased HCC risk and CLD mortality in US adults. Anti-inflammatory diets and strategies to reduce the adverse effect of hyperinsulinemia may be beneficial for prevention of HCC and reduction of CLD mortality.

## Supplementary Material

pkad023_Supplementary_Data

## Data Availability

Data used in this study are maintained by the National Cancer Institute, Division of Cancer Epidemiology and Genetics, and are available upon submitting a proposal to be approved by the NIH-AARP Steering Committee for privacy concerns. For more information visit https://www.nihaarpstars.com/.
