# Supplementary material for: Dietary inflammatory and insulinemic potential, risk of hepatocellular carcinoma, and chronic liver disease mortality
Source: JNCI Cancer Spectr. 2023 Mar 21;7(2):pkad023. doi: 10.1093/jncics/pkad023 (PMC10139765; doi:10.1093/jncics/pkad023)
Supplement: pkad023_Supplementary_Data [file pkad023_supplementary_data.pdf]

Supplementary Material

Dietary inflammatory and insulinemic potential, risk of hepatocellular carcinoma and chronic liver disease mortality

Lu Long, Xing Liu, Jessica Petrick, Wanqing Liu, Jeffrey K. Lee, Linda Liao, Michelle J. Lai, Wanshui Yang, Towia A. Libermann, Lewis R. Roberts, Katherine A. McGlynn, Fred K. Tabung, Xuehong Zhang

Supplementary Methods

- *Assessment of EDIP/EDIH*
- *Ascertainment of Covariates and Construction of Multivariable Models*
- *Definitions of HCC Cases and Liver-Related Causes of Death*

Supplementary References

Supplementary Tables

- *Supplementary table 1. Dietary components of EDIH and EDIP among NIH-AARP Diet Health Study*
- *Supplementary table 2. Age-adjusted characteristics of participants according to scores of EDIP and EDIH in women*
- *Supplementary table 3. Age-adjusted characteristics of participants according to scores of EDIP and EDIH in men*
- *Supplementary table 4. HRs and 95% CIs for HCC and CLD by quartiles of EDIP and EDIH in women*
- *Supplementary table 5. HRs and 95% CIs for HCC and CLD by quartiles of EDIP and EDIH in men*
- *Supplementary table 6. HRs and 95% CIs for the associations between EDIP/EDIH and the risk of HCC and CLD mortality after excluding cases diagnosed within the first 2 or 5 years of follow-up.*
- *Supplementary table 7. Associations between EDIP, EDIH and the risk of HCC and CLD mortality with further adjustments for BMI*

Supplementary Methods

Assessment of EDIP/EDIH

As outlined in the Methods, EDIP was derived based on 39 pre-defined food groups from FFQs using reduced-rank regression followed by stepwise linear regression models to identify a dietary pattern most predictive of 3 inflammatory biomarkers (i.e., IL-6, CRP, and TNF-alpha receptor-2). Likewise, EDIH was derived based on 39 pre-defined food groups in a separate study to identify a dietary pattern most predictive of C-peptide (an indicator of insulin secretion). A total of 18 foods and food groups were included in each dietary pattern (Supplementary Table 1).

Ascertainment of Covariates and Construction of Multivariable Models

The baseline and risk factor questionnaire contained information on a broad range of covariates, including demographic characteristics, such as age, race and ethnicity, education, lifestyle factors, such as smoking history, physical activity, alcohol intake, body weight and height; medical history such as diabetes[1]. Dietary information including total calories was obtained using a Quantitative Food Frequency Questionnaire designed for use in this multiethnic population[2].

Cox proportional hazard regression models were used to estimate hazard ratios (HRs) and 95% confidence intervals (CIs) for events comparing across categories of dietary pattern scores among men and women, respectively. Tests of linear trend across dietary score categories were conducted using the median of each category of dietary pattern scores as a continuous variable. Multivariable models included the following *a priori* covariates: age at baseline, level of education, race, BMI, alcohol use, tobacco smoking, self-reported history of diabetes, and total energy intake.

Definitions of HCC Cases and Liver-Related Causes of Death

HCC cases were identified using the International Classification of Diseases for Oncology, 3rd edition (ICD-O-3) topography (C22) and morphology codes (8170–8175 for HCC). The National Death Index Plus was used to ascertain causes of death through December 31st, 2011. Consistent with previous studies in the same cohort[3], deaths due to chronic liver diseases included deaths from liver fibrosis, cirrhosis, alcoholic liver diseases, and chronic hepatitis (ICD-9: 571.0, 571.2–571.6, 571.8, and 571.9; ICD-10: K70, K73, and K74). Liver cancer deaths were not counted in CLD deaths.

Supplementary References:

1. Schatzkin A, Subar AF, Thompson FE, et al. Design and serendipity in establishing a large cohort with wide dietary intake distributions : the National Institutes of Health-American Association of Retired Persons Diet and Health Study. Am J Epidemiol 2001;154:1119-25.
2. Kolonel LN, Henderson BE, Hankin JH, et al. A multiethnic cohort in Hawaii and Los Angeles: baseline characteristics. Am J Epidemiol 2000;151:346-57.
3. Li WQ, Park Y, McGlynn KA, et al. Index-based dietary patterns and risk of incident hepatocellular carcinoma and mortality from chronic liver disease in a prospective study. Hepatology 2014;60:588-97.

Supplementary Table 1. Dietary components of EDIH and EDIP among NIH-AARP Diet Health Study

	Positive associations	Inverse associations
**EDIP**	Processed meat	Beer
	Red meat	Wine
	Organ meat	Tea
	Other fish	Coffee
	Other vegetables	Dark yellow vegetables
	Refined grains	Leafy green vegetables
	High-energy beverages	Snacks
	Low-energy beverages	Fruit juice
	Tomatoes	Pizza
**EDIH**	Red meat	Wine
	Low-energy beverages	Coffee
	Cream soups	Whole fruits
	Processed meat	High-fat dairy products
	Margarine	Leafy green vegetables
	Poultry	
	Butter	
	French fries	
	Other fish	
	High-energy beverages	
	Tomatoes	
	Low-fat dairy products	
	Eggs	

Supplementary Table 2. Age-adjusted characteristics of participants according to scores of EDIP and EDIH in women

	EDIP				EDIH			
	Quartile 1 (n=54583)	Quartile 2 (n=53240)	Quartile 3 (n=48527)	Quartile 4 (n=38960)	Quartile 1 (n=52891)	Quartile 2 (n=56576)	Quartile 3 (n=49690)	Quartile 4 (n=36153)
Age at baseline*	61.3 (5.4)	61.7 (5.3)	61.6 (5.4)	60.7 (5.5)	61.6 (5.4)	61.7 (5.3)	61.4 (5.4)	60.4 (5.5)
White, %	92.7	92.5	89.8	88.0	90.8	91.1	90.8	91.6
College education, %	36.2	31.8	29.1	24.9	37.7	32.3	28.0	23.3
BMI, kg/m^2	25.9 (5.5)	26.4 (5.7)	27 (6)	28.4 (6.6)	25.5 (5.3)	26.3 (5.6)	27.3 (6.1)	28.8 (6.7)
Physical activity at least 20 minutes, ≥5 times/week, %	20.7	16.2	14.4	12.9	22.6	16.4	13.3	11.1
Alcohol, drinking/week	4.3 (8.8)	2.7 (6.2)	2.1 (6.1)	1.8 (7.2)	4.8 (10)	2.5 (6.3)	1.8 (5.1)	1.9 (5.6)
Current smoking, %	17.1	14.8	12.4	13.4	14.2	13.8	13.7	17.5
History of diabetes, %	5.1	5.8	7.5	12.4	4.0	5.5	8.2	14.2
Aspirin use, %	67.3	67.3	65.3	62.3	67.0	66.7	65.2	63.4
Total energy intake, kcal/d	1626 (572)	1483 (542)	1457 (552)	1605 (629)	1734 (578)	1466 (520)	1383 (538)	1590 (618)

EDIP, Empirical dietary inflammatory pattern; EDIH, Empirical dietary index for hyperinsulinemia.

Values are means (SD) or medians (Q25, Q75) for continuous variables; percentages or both for categorical variables, and are standardized to the age distribution of the study population.

* Value is not age adjusted

Supplementary Table 3. Age-adjusted characteristics of participants according to scores of EDIP and EDIH in men

	EDIP				EDIH			
	Quartile 1 (n=66875)	Quartile 2 (n=70439)	Quartile 3 (n=73553)	Quartile 4 (n=79754)	Quartile 1 (n=67766)	Quartile 2 (n=67886)	Quartile 3 (n=71432)	Quartile 4 (n=83537)
Age at baseline*	61.5 (5.3)	61.8 (5.3)	62 (5.3)	61.2 (5.4)	62 (5.2)	62.1 (5.3)	61.8 (5.3)	60.9 (5.4)
White, %	95.1	95.1	93.8	92.0	93.8	93.5	93.8	94.5
College education, %	51.8	48.0	44.7	39.4	54.3	48.7	44.3	37.4
BMI, kg/m^2	26.8 (4)	27 (4.1)	27.2 (4.2)	27.8 (4.7)	26.4 (3.8)	26.8 (4)	27.3 (4.1)	28.2 (4.7)
Physical activity at least 20 minutes, ≥5 times/week, %	25.3	21.6	20.0	19.4	28.5	22.2	18.6	17.4
Alcohol, drinking/week	11.8 (18.7)	7.4 (12.4)	5.9 (11.9)	4.8 (12.6)	12.3 (20.1)	7.2 (13.2)	5.7 (11.1)	4.9 (10.5)
Current smoking, %	12.1	10.9	9.9	9.9	9.2	10.0	10.6	12.3
History of diabetes, %	7.7	8.1	9.6	14.4	5.7	7.3	9.5	16.7
Aspirin use, %	79.8	79.7	78.4	75.8	79.3	79.1	78.3	76.9
Total energy intake, kcal/d	2068 (682)	1893 (639)	1858 (640)	2045 (709)	2153 (675)	1865 (625)	1805 (635)	2033 (701)

EDIP, Empirical dietary inflammatory pattern; EDIH, Empirical dietary index for hyperinsulinemia.

Values are means (SD) or medians (Q25, Q75) for continuous variables; percentages or both for categorical variables, and are standardized to the age distribution of the study population.

* Value is not age adjusted

Supplementary Table 4. HRs and 95% CIs for HCC and CLD by quartiles of EDIP and EDIH in women

	HR (95% CI)				
	**Quartile 1**	**Quartile 2**	**Quartile 3**	**Quartile 4**	P_{trend}[a]
			**EDIP**		
**HCC**					
Number of cases	26	25	30	35	
Age-adjusted model*	1 (Ref)	0.97 (0.56-1.67)	1.28 (0.76-2.17)	2.04 (1.23-3.39)	0.003
Model 1**	1 (Ref)	1.11 (0.62-1.98)	1.41 (0.80-2.46)	2.17 (1.27-3.71)	0.003
Model 2***	1 (Ref)	1.09 (0.61-1.93)	1.31 (0.75-2.29)	1.78 (1.03-3.06)	0.03
**CLD mortality**					
Number of cases	60	67	80	94	
Age-adjusted model*	1 (Ref)	1.13 (0.80-1.60)	1.49 (1.07-2.08)	2.32 (1.67-3.20)	<0.0001
Model 1**	1 (Ref)	1.36 (0.94-1.94)	1.73 (1.22-2.47)	2.46 (1.74-3.47)	<0.0001
Model 2***	1 (Ref)	1.33 (0.93-1.91)	1.62 (1.14-2.31)	2.03 (1.43-2.89)	<0.0001
			**EDIH**		
**HCC**					
Number of cases	22	34	30	30	
Age-adjusted model*	1 (Ref)	1.45 (0.85-2.47)	1.50 (0.87-2.60)	2.29 (1.32-3.97)	0.005
Model 1**	1 (Ref)	1.91 (1.07-3.39)	1.75 (0.95-3.23)	2.89 (1.60-5.22)	0.01
Model 2***	1 (Ref)	1.81 (1.02-3.22)	1.52 (0.82-2.82)	2.17 (1.18-3.97)	0.03
**CLD mortality**					
Number of cases	64	76	76	85	
Age-adjusted model*	1 (Ref)	1.11 (0.80-1.55)	1.30 (0.93-1.81)	2.13 (1.54-2.95)	<0.0001
Model 1**	1 (Ref)	1.31 (0.92-1.86)	1.45 (1.00-2.08)	2.41 (1.70-3.41)	<0.0001
Model 2***	1 (Ref)	1.26 (0.88-1.79)	1.29 (0.89-1.86)	1.87 (1.30-2.67)	0.001

HR, Hazard ratio; CI, Confidence interval; EDIP, Empirical dietary inflammatory pattern; EDIH, Empirical dietary index for hyperinsulinemia.

* Adjusted for age (in years).

**Adjusted for age (in years), gender (women, men), race (white, non-white), education (≤11 yrs, high school, vocational technology school, some college, college/post graduate), physical activity (never, rarely, 1-3 times/month, 1-2 times/week, 3-4 times/week, ≥5 times/week), smoking status (never, past, current), aspirin use (yes, no), alcohol intake (g/day, continuous) and total calorie intake (kcal/day, continuous).

***Adjusted for covariates in Model 1 plus history of diabetes (yes, no).

[a] The p-value for linear trend was obtained using EDIP or EDIH quartile medians as an ordinal variable adjusted for the covariates listed above.

Supplementary Table 5. HRs and 95% CIs for HCC and CLD by quartiles of EDIP and EDIH in men

	HR (95% CI)				
	**Quartile 1**	**Quartile 2**	**Quartile 3**	**Quartile 4**	P_{trend}[a]
			**EDIP**		
**HCC**					
Number of cases	108	91	145	175	
Age-adjusted model*	1 (Ref)	0.79 (0.60-1.05)	1.21 (0.94-1.56)	1.42 (1.11-1.80)	0.0001
Model 1**	1 (Ref)	0.85 (0.64-1.13)	1.26 (0.97-1.63)	1.39 (1.08-1.80)	0.0006
Model 2***	1 (Ref)	0.85 (0.64-1.13)	1.23 (0.95-1.59)	1.23 (0.96-1.59)	0.01
**CLD mortality**					
Number of cases	141	149	170	232	
Age-adjusted model*	1 (Ref)	0.99 (0.79-1.25)	1.09 (0.87-1.36)	1.43 (1.16-1.77)	0.0002
Model 1**	1 (Ref)	1.15 (0.91-1.45)	1.24 (0.99-1.57)	1.65 (1.33-2.05)	<0.0001
Model 2***	1 (Ref)	1.14 (0.90-1.44)	1.21 (0.96-1.52)	1.52 (1.22-1.89)	0.0002
			**EDIH**		
**HCC**					
Number of cases	94	105	139	181	
Age-adjusted model*	1 (Ref)	1.13 (0.85-1.49)	1.46 (1.12-1.89)	1.73 (1.35-2.22)	<0.0001
Model 1**	1 (Ref)	1.20 (0.90-1.60)	1.50 (1.14-1.98)	1.72 (1.32-2.24)	<0.0001
Model 2***	1 (Ref)	1.16 (0.87-1.55)	1.37 (1.03-1.80)	1.34 (1.03-1.76)	0.02
**CLD mortality**					
Number of cases	129	157	173	233	
Age-adjusted model*	1 (Ref)	1.22 (0.97-1.54)	1.32 (1.05-1.65)	1.61 (1.30-2.00)	<0.0001
Model 1**	1 (Ref)	1.44 (1.13-1.84)	1.62 (1.27-2.07)	1.83 (1.45-2.31)	<0.0001
Model 2***	1 (Ref)	1.41 (1.11-1.81)	1.54 (1.21-1.97)	1.59 (1.25-2.01)	0.0002

HR, Hazard ratio; CI, Confidence interval; EDIP, Empirical dietary inflammatory pattern; EDIH, Empirical dietary index for hyperinsulinemia.

* Adjusted for age (in years).

**Adjusted for age (in years), gender (women, men), race (white, non-white), education (≤11 yrs, high school, vocational technology school, some college, college/post graduate), physical activity (never, rarely, 1-3 times/month, 1-2 times/week, 3-4 times/week, ≥5 times/week), smoking status (never, past, current), aspirin use (yes, no), alcohol intake (g/day, continuous), and total calorie intake (kcal/day, continuous).

***Adjusted for covariates in Model 1 plus history of diabetes (yes, no).

[a] The p-value for linear trend was obtained using EDIP or EDIH quartile medians as an ordinal variable adjusted for the covariates listed above.

Supplementary Table 6. HRs and 95% CIs for the associations between EDIP/EDIH and the risk of HCC and CLD mortality after excluding cases diagnosed within the first 2 or 5 years of follow-up

	HR (95% CI)				
	**Quartile 1**	**Quartile 2**	**Quartile 3**	**Quartile 4**	***P*$_{trend}$[a]**
**EDIP**					
**HCC**					
2 years excluded (n=633)	1 (Ref)	0.87 (0.68-1.13)	1.24 (0.98-1.57)	1.36 (1.08-1.71)	0.0005
5 years excluded (n=629)	1 (Ref)	0.87 (0.68-1.13)	1.24 (0.98-1.57)	1.36 (1.08-1.71)	0.0007
**CLD mortality**					
2 years excluded (n=986)	1 (Ref)	1.20 (0.98-1.46)	1.35 (1.11-1.63)	1.68 (1.40-2.02)	<0.0001
5 years excluded (n=975)	1 (Ref)	1.19 (0.98-1.45)	1.33 (1.10-1.62)	1.66 (1.37-1.99)	<0.0001
**EDIH**					
**HCC**					
2 years excluded (n=633)	1 (Ref)	1.37 (1.07-1.75)	1.52 (1.19-1.94)	1.23 (0.95-1.59)	0.0004
5 years excluded (n=629)	1 (Ref)	1.21 (0.93-1.56)	1.37 (1.07-1.75)	1.52 (1.19-1.94)	0.0005
**CLD mortality**					
2 years excluded (n=986)	1 (Ref)	1.40 (1.14-1.71)	1.51 (1.24-1.85)	1.72 (1.41-2.09)	<0.0001
5 years excluded (n=975)	1 (Ref)	1.40 (1.14-1.71)	1.47 (1.20-1.80)	1.70 (1.40-2.07)	<0.0001

HR, Hazard ratio; CI, Confidence interval; EDIP, Empirical dietary inflammatory pattern; EDIH, Empirical dietary index for hyperinsulinemia.

Model adjusted for age (in years), gender (women, men), race (white, non-white), education (≤11 yrs, high school, vocational technology school, some college, college/post graduate), physical activity (never, rarely, 1-3 times/month, 1-2 times/week, 3-4 times/week, ≥5 times/week), smoking status (never, past, current), aspirin use (yes, no), alcohol intake (g/day, continuous), total calorie intake (kcal/day, continuous) and history of diabetes (yes, no).

[a] The p-value for linear trend was obtained using EDIP or EDIH quartile medians as an ordinal variable adjusted for the covariates listed above.

Supplementary Table 7. Associations between EDIP, EDIH and the risk of HCC and CLD mortality with further adjustments for BMI and mutually adjustment

	HR (95% CI)				
	Quartile 1	Quartile 2	Quartile 3	Quartile 4	P_{trend}[a]
**EDIP**					
**HCC**					
BMI adjusted model*	1 (Ref)	0.85 (0.66-1.10)	1.22 (0.97-1.55)	1.25 (1.00-1.58)	0.002
Mutually adjusted model**	1 (Ref)	0.78 (0.60-1.02)	1.02 (0.78-1.34)	1.06 (0.79-1.44)	0.002
**CLD mortality**					
BMI adjusted model*	1 (Ref)	1.12 (0.92-1.37)	1.20 (0.99-1.46)	1.46 (1.21-1.77)	<0.0001
Mutually adjusted model**	1 (Ref)	1.10 (0.89-1.35)	1.17 (0.94-1.46)	1.45 (1.14-1.86)	0.003
**EDIH**					
**HCC**					
BMI adjusted model*	1 (Ref)	1.21 (0.94-1.57)	1.35 (1.05-1.74)	1.42 (1.11-1.81)	0.004
Mutually adjusted model**	1 (Ref)	1.23 (0.97-1.61)	1.39 (1.08-1.84)	1.59 (1.24-1.93)	0.002
**CLD mortality**					
BMI adjusted model*	1 (Ref)	1.17 (0.95-1.43)	1.23 (1.01-1.50)	1.31 (1.08-1.58)	0.007
Mutually adjusted model**	1 (Ref)	1.27 (1.02-1.56)	1.31 (1.04-1.65)	1.50 (1.16-1.93)	0.004

HR, Hazard ratio; CI, Confidence interval; EDIP, Empirical dietary inflammatory pattern; EDIH, Empirical dietary index for hyperinsulinemia.

* Adjusted for covariates in Model 2 of Table 2 plus body mass index (<25, 25-29.9, ≥30, kg/m^2).

** Mutually adjustment for EDIP and EDIH in the same model.

[a] The p-value for linear trend was obtained using EDIP or EDIH quartile medians as an ordinal variable adjusted for the covariates listed above.
